# Supplementary material for: Posiphen Reduces the Levels of Huntingtin Protein through Translation Suppression
Source: Pharmaceutics. 2021 Dec 7;13(12):2109. doi: 10.3390/pharmaceutics13122109 (PMC8708689; doi:10.3390/pharmaceutics13122109)
Supplement: Supplementary file 1 [file pharmaceutics-13-02109-s001.zip › Supplementary Materials Figure S1 Posiphen reduced HTT levels in human fibroblast and pluripotent human stem cell lines.pdf]

pharmaceutics

Supplementary Materials: Posiphen Reduces the Levels of Huntingtin Protein Through Translation Suppression

Xu-Qiao Chen, Carlos A. Barrero, Rodrigo Vasquez-Del Carpio, E. Premkumar Reddy, Chiara Fecchio, Salim Merali, Alessia Deglincerti, Cheng Fang, Jack Rogers and Maria L. Maccecchini

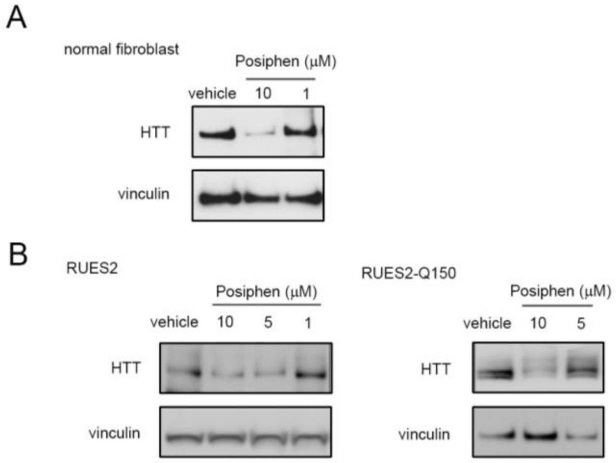

Figure S1. Posiphen reduced HTT levels in human fibroblast and pluripotent human stem cell lines. (**A**) Wild type human fibroblasts and (**B**) the WT-RUES2 and RUES2-Q150 pluripotent human stem cell lines were treated with different concentrations of Posiphen as indicated for 48 hours, followed by immunoblotting to analyze the levels of HTT. Vinculin served as an internal loading control.
